# Blend-Brewing Optimization of *Ganoderma lucidum* Yellow Tea: Improving Palatability by Regulating Compound Release and Nanoparticle Assembly

**DOI:** 10.3390/foods15162876

**Published:** 2026-08-17

**Authors:** Anran Yan, Weiyi Kong, Xinyu Feng, Ning Tang, Fangyuan Fan, Ping Chen, Binggan Lou, Qiang Chu, Ruili Zhang

**Affiliations:** 1 Tea Research Institute, Zhejiang University, Hangzhou 310058, China; 2 State Key Laboratory of Rice Biology and Breeding, Ministry of Agriculture Key Laboratory of Molecular Biology of Crop Pathogens and Insects, Institute of Biotechnology, Zhejiang University, Hangzhou 310058, China; 3 Xinjiang Production & Construction Group Key Laboratory of Agricultural Products Processing in Xinjiang South, College of Food Science and Engineering, Tarim University, Alar 843300, China

**Keywords:** flavored tea, tea quality, nanoparticles, tea chemistry, *Ganoderma lucidum*

## Abstract

Flavored teas have gained popularity because they combine sensory appeal with potential health benefits. *Ganoderma lucidum* (GL) is a promising functional beverage ingredient owing to its high medicinal value. However, its strong fungal off-flavor and bland taste limit consumer acceptance. Yellow tea (YT), characterized by a mild-sweet aroma and mellow mouthfeel developed during sealed yellowing, provides a suitable matrix for moderating the sensory defects of GL. This study designed a blend-brewing strategy, optimizing the YT to GL ratio (2:3, *w*/*w*) and comparing covered (CB), standard (SB), and open-topped brewing (OB) methods. SB achieved the most balanced sensory profile. In contrast, CB intensified bitterness and astringency, which were associated with higher concentrations of EGCG, gallic acid, and theobromine, whereas OB produced a relatively flat taste because of insufficient extraction. Brewing also reshaped self-assembled nanoparticles, with CB inducing pronounced aggregation and SB yielding a broader size distribution, smoother surfaces, and more balanced colloidal proteins, catechins, and caffeine. Removing nanoparticles substantially decreased infusion mellowness, supporting a contribution of the NP-enriched fraction to mouthfeel. These findings demonstrate that *Ganoderma lucidum* yellow tea (GY) blending and brewing method modulate sensory quality through chemical extraction and colloidal nanoparticle organization, providing a practical strategy to improve *Ganoderma lucidum* palatability and develop functional tea beverages.

## 1. Introduction

Flavored tea (FT) combines tea (*Camellia sinensis*) with fruits, herbs, flowers, or other ingredients to create beverages with differentiated sensory profiles [1]. Appropriate blending and brewing could mask off-flavors and highlight preferable flavors, thereby improving overall palatability and consumer acceptance. *Ganoderma lucidum* (GL), a traditional Chinese medicinal mushroom with reported antitumor and immunomodulatory properties, has been increasingly incorporated into foods and beverages, while its fungal flavor and insipid taste limit sensory acceptance [2,3]. Yellow tea (YT), whose sealed-yellowing process produces a mild-sweet aroma and mellow taste [4], offers a complementary matrix for moderating GL-derived off-notes. Pairing GL with YT is therefore a promising route to improve palatability [1,5].

From raw materials to the final cup, formulation and brewing are among the last and most influential steps in beverage preparation. Blending can create a balance of taste, aroma, and appearance that may not be achievable with a single ingredient, while the blend ratio determines the relative contributions of complementary and competing sensory attributes [6]. A previous study optimized the blending ratio of coffee, tea and Rooibos to mitigate the burnt bitterness [1]. Brewing subsequently determines how the formulated matrix is expressed by regulating solute extraction, volatile release, evaporation, and headspace exchange [7]. Brewing methods influence the FT sensory quality comprehensively from dimensions of extraction rate temperature maintenance and headspace pressure. In the field of coffee brewing, a novel espresso brewing method that introduced pressurized air into the chamber led to higher viscosity and strengthened the tactile sensation in the mouth [8]. Gaiwan and French press brewing strategies selectively enhanced the phenolic and flavonoids extraction rate, while the pure brewing method strengthened aroma sweetness and aromatic clarity [9]. Current research showed that top-brewing (putting tea into water) promoted the leaching of phenolic compounds and sustained the molecular structure of EGCG, and therefore presented more intense bitterness than bottom-brewing (pouring water onto tea) methods [10]. Heating can further promote transformations such as theanine-glucose Maillard reactions, catechin degradation and isomerization [11], degradation and isomerization of epi-catechins [5], and thus reshaping the sensory profiles of brewing infusion. These observations indicate that formulation and brewing are interdependent processes that jointly shape the chemical substances and sensory characteristics of the final infusion.

Tea infusion is also a complex dispersion rather than a true solution. Brewing can redistribute flavor-related constituents between the continuous and colloidal phases [12]. Spontaneously assembled infusion nanoparticles (NPs) contain polyphenols, alkaloids, proteins, and polysaccharides and form through noncovalent interactions, including hydrogen bonding, hydrophobic interactions, and electrostatic forces [13]. Therefore, the chemical basis of liquid and brewing conditions further sculptures the physicochemical properties of infusion, which also plays a nonnegligible role in the mouthfeel and taste of infusion. For instance, the increased degree of NP elimination from black tea led to a gradual decline of sweetness and thickness, and impaired the final sensory scores of infusion [14]. In another food matrix, the addition of a mushroom ingredient altered the dispersion and emulsification behavior of micro- and nanoparticles in chicken soup [15]. Model-food studies have further shown that increasing the size of silica microparticles can intensify perceived roughness [16]. Similarly, the modulus of agar microparticles increased the chalkiness mouthcoating attributes [17]. Together, these findings suggest that brewing-dependent changes in colloidal organization, as well as changes in dissolved composition, may contribute to beverage mouthfeel and taste.

Despite these advances, formulation, brewing, and colloidal behavior have generally been investigated as separate aspects of beverage quality. Studies of blended beverages have mainly optimized ingredient ratios or masked undesirable notes [1,6], whereas brewing studies have focused on volatile release, phenolic extraction, or sensory changes in conventional tea and related infusions [7,8,9,18]. In parallel, research on self-assembled infusion particles has centered on single-tea systems [12,14]. It therefore remains unclear How co-brewing a medicinal mushroom-tea matrix simultaneously reshapes the dissolved and colloidal phases, and how those phase-level changes are associated with integrated sensory perception. This gap matters because blend composition defines the pool of flavor-active and particle-forming precursors, whereas brewing governs their extraction, transformation, and partitioning.

In this study, we designed a novel blending-brewing method to acquire *Ganoderma lucidum* yellow tea (GY) with coordinated and ample sensory characteristics. After establishment of the appropriate blending ratio, electronic tongue, scanning electron microscope, high performance liquid chromatography and other physicochemical analysis were conducted to investigate the chemical profile and physical properties of liquid and colloids phase. The novelty of this study lies not simply in identifying a preferred blend ratio or brewing protocol, but in integrating sensory perception with chemical extraction and colloidal organization in a co-brewed medicinal mushroom-tea system. This integrated sensory, chemical, and colloidal analysis provides a useful reference for flavor assessment and formulation development of flavored tea beverages.

## 2. Materials and Methods

### 2.1. Materials and Reagents

*Ganoderma lucidum* (GL) was purchased from Jiayin Trading Co., Ltd. (Lishui, China). Yellow tea (YT) was obtained from Panyue Tea Industry Co., Ltd. (Huzhou, China). All samples were stored under dry and dark conditions until use. Catechin standards, including catechin (C), gallocatechin (GC), catechin gallate (CG), gallocatechin gallate (GCG), epicatechin (EC), epigallocatechin (EGC), epicatechin gallate (ECG), and epigallocatechin gallate (EGCG), as well as gallic acid, caffeine, and theobromine standards, were of chromatographic grade (purity > 99%) and were purchased from Aladdin Biochemical Technology Co., Ltd. (Shanghai, China). Analytical-grade acetonitrile, anhydrous glucose, aluminum nitrate nonahydrate, guanidine hydrochloride, and acetic acid were obtained from Traditional Chinese Medicine Chemical Reagent Co., Ltd. (Shanghai, China).

### 2.2. Preparation of Ganoderma lucidum Yellow Tea Blends

*Ganoderma lucidum* yellow tea (GY) samples were prepared by blending YT with GL at different ratios. YT and GL were used as control groups, whereas GY-1 to GY-6 represented blends with YT:GL mass ratios of 3:1, 2:1, 3:2, 2:3, 1:2, and 1:3, respectively. After thorough mixing and brewing with national standard method (GB/T 23776-2018) [19], all samples were subjected to sensory evaluation, and the optimal blending ratio was selected based on infusion color, aroma, taste, and total sensory score. According to the sensory evaluation results, GY samples with the optimal blending ratio were further used to compare the effects of brewing methods. GY samples were brewed using three protocols: covered brewing (CB), standard brewing according to GB/T 23776-2018 (SB), and open-topped brewing (OB). YT and GL were still used as control groups. For CB, the infusion vessel was covered throughout brewing to maintain heat and limit gas exchange. For SB, samples were brewed according to GB/T 23776-2018. For OB, the vessel remained open during the entire brewing process to allow greater heat dissipation and gas exchange. For all treatments, 3.0 g of each sample was brewed with 150 mL of water for 5 min. The brewing vessel was immersed in a 100 °C water bath throughout extraction, and the initial and final infusion temperatures were both 100 °C.

### 2.3. Sensory Evaluation and Quantitative Descriptive Analysis

Sensory evaluation was conducted in two stages. Blends with different YT:GL ratios were first evaluated according to GB/T 23776-2018. QDA was subsequently performed with reference to ASTM MNL 13 because it provides established guidance on sensory-attribute development, intensity scaling, and quantitative descriptive evaluation [20]. Specifically, seven trained panelists (three men and four women) with more than three years of sensory-evaluation experience participated. In accordance with ISO 8586:2023 [21], their sensory ability was evaluated by basic-taste identification, odor recognition, and intensity-ranking tests. Panel discrimination, repeatability, and agreement were evaluated using replicate samples according to ISO 11132:2021 [22]. Evaluations were conducted in a sensory room under ISO 8589:2007 [23] conditions at 24 ± 1 °C and 65 ± 2% relative humidity, under white light and without extraneous odors or noise. Tea infusions were prepared using commercially available purified water supplied in 19 L bottles (Wahaha, Hangzhou, China) at a tea-to-water ratio of 1:50 (*w*/*v*). The samples were infused with boiling water for 5 min and presented immediately in identical standard cylindrical tea-tasting cups specified in GB/T 23776-2018. Each sample was labeled with a random three-digit code and presented in a randomized order. Blend screening used a separate 100-point system for infusion color, aroma, and taste, weighted 15%, 40% and 45%, respectively. The six QDA attributes were sweetness, umami, bitterness, astringency, fungal, and mellowness and were rated from 0 (not perceptible) to 5 (very strong). Each sample was evaluated in three independent sessions.

### 2.4. Determination of Infusion Color Parameters

Filtered tea infusions were transferred into transparent cuvettes and analyzed using a CM-5 colorimeter (Konica Minolta, Tokyo, Japan) over 380–780 nm. The measured parameters included L*, a*, and b*. L* represents lightness, a* represents the red-green axis (+/−), and b* represents the yellow-blue axis (+/−) [24]. Chroma (C*), hue angle (*h*°), and total color difference (Δ*E*) were calculated as follows:

$$C^{*}=\sqrt{(a^{*}{)}^{2}+(b^{*}{)}^{2}}$$

$$h^{\circ }=atan2(b^{*},a^{*})\times \frac{180^{\circ }}{\pi }$$

$$\Delta E=\sqrt{(L_{i}^{*}-L_{s}^{*}{)}^{2}+(a_{i}^{*}-a_{s}^{*}{)}^{2}+(b_{i}^{*}-b_{s}^{*}{)}^{2}}$$

where (i) denotes the covered-brewing or open-topped-brewing sample, and (s) denotes the corresponding infusion prepared using the standard brewing method. For negative $\left(h^{\circ }\right)$ values, 360° was added to obtain a hue angle ranging from 0° to 360°. For each raw material (YT, GL, and GY), the infusion prepared using the standard brewing method was used as the reference for calculating (ΔE). Each sample was measured in triplicate.

### 2.5. Electronic Tongue Analysis

Filtered tea infusions were cooled to 25 ± 1 °C, and their pH values 6.5 were recorded. Because the samples were filtered tea infusions, no additional homogenization was performed. A 25 mL infusion was analyzed using an electronic tongue (Alpha MOS, Toulouse, France) equipped with seven cross-sensitive potentiometric sensors (AHS, PKS, CTS, NMS, CPS, ANS, and SCS) and an Ag/AgCl reference electrode. Before analysis, the sensors were conditioned and calibrated with 0.01 M HCl and diagnosed using 0.01 M HCl, NaCl, and monosodium glutamate according to the procedure of the manufacturer. Potentiometric signals were acquired at 1 s intervals for 120 s using AlphaSoft v16.0 (Alpha MOS, Toulouse, France), and the mean stable response from 100 to 120 s was used. Between measurements, the sensors were rinsed with deionized water for 10 s until the baseline was restored [25]. Six replicate measurements were performed for each sample.

### 2.6. Isolation and Preparation of Colloidal Particles from Tea Infusions

A 50 mL aliquot of tea infusion was centrifuged at 12,000 rpm for 20 min at 4 °C. The supernatant was discarded, and the precipitate was resuspended in ultrapure water. The suspension was then centrifuged at 6000 rpm for 15 min at 4 °C, and the supernatant was collected. This washing and resuspension procedure was repeated twice to obtain colloidal particles [14].

### 2.7. Analysis of Catechins and Alkaloids

Catechins and alkaloids were determined using an HPLC-UV system (Shimadzu, Kyoto, Japan) equipped with an Agilent TC-C18 column (4.6 × 250 mm, 5 μm). For catechins and alkaloids, mobile phases A and B consisted of acetonitrile, acetic acid, and water at ratios of 6:1:193 and 60:1:139 (*v*/*v*/*v*), respectively. The gradient was programmed as follows: 20–75% B from 0 to 40 min, 75–20% B from 40 to 45 min, and 20% B from 45 to 50 min, with detection at 280 nm [12,26].

### 2.8. Determination of Flavonoids, Tea Polyphenols, Proteins, Soluble Sugars, Triterpenoids, and Free Amino Acids

Total flavonoid content was determined using the sodium nitrite–aluminum nitrate colorimetric method with rutin as the standard, and absorbance was recorded at 510 nm. Total polyphenol content was determined using the Folin–Ciocalteu method with gallic acid as the standard, and absorbance was recorded at 765 nm. Protein content was determined using the Coomassie Brilliant Blue method with bovine serum albumin (BSA) as the standard, and absorbance was recorded at 595 nm. Soluble sugar content was determined using the anthrone-sulfuric acid colorimetric method with glucose as the standard, and absorbance was recorded at 620 nm. Total triterpenoid content was determined using the vanillin-perchloric acid colorimetric method with ursolic acid as the standard, and absorbance was recorded at 546 nm. Free amino acid content was determined using the ninhydrin colorimetric method, and absorbance was measured at 570 nm [27,28]. All measurements were performed in triplicate.

### 2.9. Scanning Electron Microscopy

An appropriate amount of colloidal particle suspension was deposited onto a silicon wafer, naturally dried, and sputter-coated with gold. Particle morphology was then examined using a scanning electron microscopy (SEM, Zeiss, Oberkochen, Germany) [27].

### 2.10. UV-Visible Absorption Spectroscopy

Colloidal particles were appropriately diluted before UV-visible (UV-vis) spectroscopic analysis. Ultrapure water was used as the blank control. Absorption spectra were recorded from 200 to 600 nm, with particular attention to absorbance intensity in the 200–250 nm range [29]. Each sample was measured in triplicate.

### 2.11. Determination of Zeta Potential and Particle Size Distribution

Zeta potential and particle size distribution were measured using dynamic light scattering (Malvern, Shanghai, China). Samples were diluted with ultrapure water before measurement to avoid multiple scattering. Measurements were performed at 25 °C. Zeta potential, expressed in mV, was used to characterize the surface charge of colloidal particles. Particle size distribution was expressed as intensity distribution and used to compare particle size and distribution range among samples [30]. Each sample was measured in triplicate.

### 2.12. Data Processing and Statistical Analysis

Statistical analyses were performed using IBM SPSS Statistics 26.0. Normality and homogeneity of variance were assessed using the Shapiro–Wilk and Levene’s tests, respectively. All variables satisfied the normality assumption. Homogeneity of variance was satisfied for all variables except for the total polyphenol content of tea infusions (Levene’s F = 8.302, *p* = 0.003). Therefore, one-way ANOVA followed by Tukey’s test was used for variables meeting both assumptions. Tukey’s test was selected because all pairwise group comparisons were required while controlling the family-wise error rate. The Brown–Forsythe test followed by the Games–Howell test was used for total polyphenol content because of unequal variances. *p* < 0.05 was considered statistically significant.

## 3. Results and Discussion

### 3.1. Effects of Blending Ratio on the Sensory Quality of Ganoderma lucidum Yellow Tea

**Figure 1:**
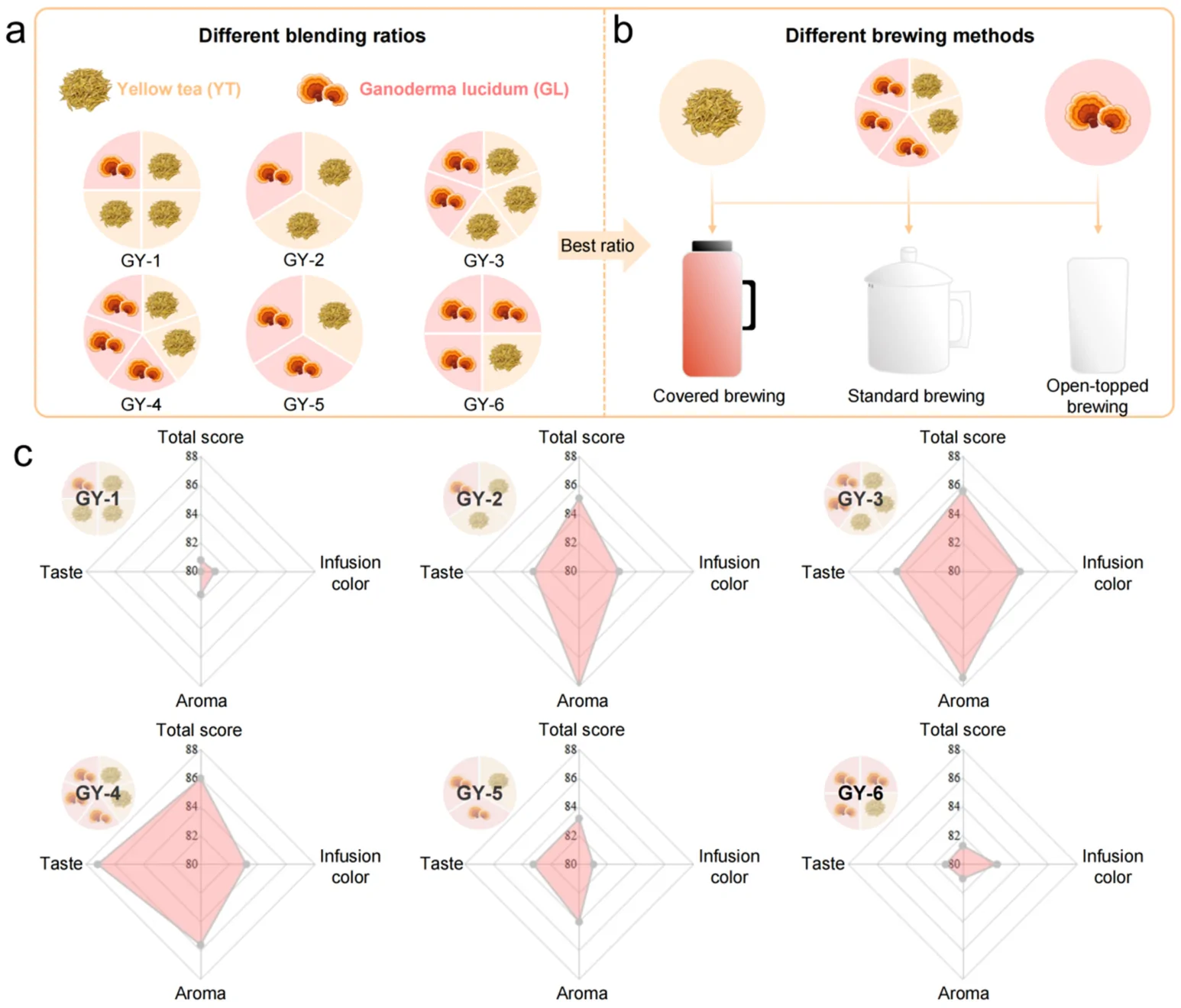
Blend-ratio screening and brewing-method optimization of GY. (**a**) Screening of blends with different YT and GL ratios; (**b**) comparison of brewing methods after selection of the optimal blending ratio; (**c**) sensory evaluation scores of different blending ratios. Sensory scores are presented as the mean values obtained from seven panelists (*n* = 7). YT, yellow tea; GL, *Ganoderma lucidum*; GY, *Ganoderma lucidum* yellow tea; GY-1-GY-6, *Ganoderma lucidum* yellow tea with different blending ratios.

The blending ratio is critical for flavored teas; for instance, the tea-to-milk ratio has been reported as the most important factor affecting flavor [31]. This study preliminarily evaluated the sensory quality of the GY blending samples through different YT:GL ratios. According to the overall design shown in Figure 1a,b, samples with different GY ratios were first compared in terms of infusion color, aroma, taste, and total score to identify the blend with the best flavor coordination. This selected blend was then used to evaluate the effects of brewing method. Therefore, this section focuses on blend optimization, whereas the systematic comparison of brewing methods is presented in Section 3.2. Sensory evaluation showed that GY integrated the mellow taste of YT with the GL characteristic aroma, indicating that blending partially compensated for the weak taste and overly strong fungal note of GL. The blending ratio markedly affected overall sensory quality (Table 1). Among all blends, GY-4 (YT:GL = 2:3) obtained the highest total score (86.0, Figure 1c), with a relatively bright orange-yellow infusion, a distinct GL aroma accompanied by tea and sweet notes, and a sweet, mellow, slightly umami, and harmonious taste. These results suggested that this ratio provided the most suitable balance between YT-derived and GL-derived flavor attributes. GY-2 (2:1) and GY-3 (3:2) also received relatively high sensory scores, implying that moderate GL addition improved aroma complexity and taste harmony. However, further increasing the proportion of GL in GY-5 (1:2) and GY-6 (1:3) intensified the fungal aroma while masking the tea-derived flavor, which reduced the total score. These findings indicated that excessive GL addition could not achieve a simultaneous improvement in both aroma complexity and taste coordination. Collectively, an appropriate GL proportion enhanced flavor richness, whereas excessive addition impaired overall coordination. For the selected 2:3 blend, standard brewing maintained the characteristic GL aroma while balancing sweetness, mellowness, bitterness, and astringency.

### 3.2. Effects of Brewing Method on the Quality of Ganoderma lucidum Yellow Tea

**Figure 2:**
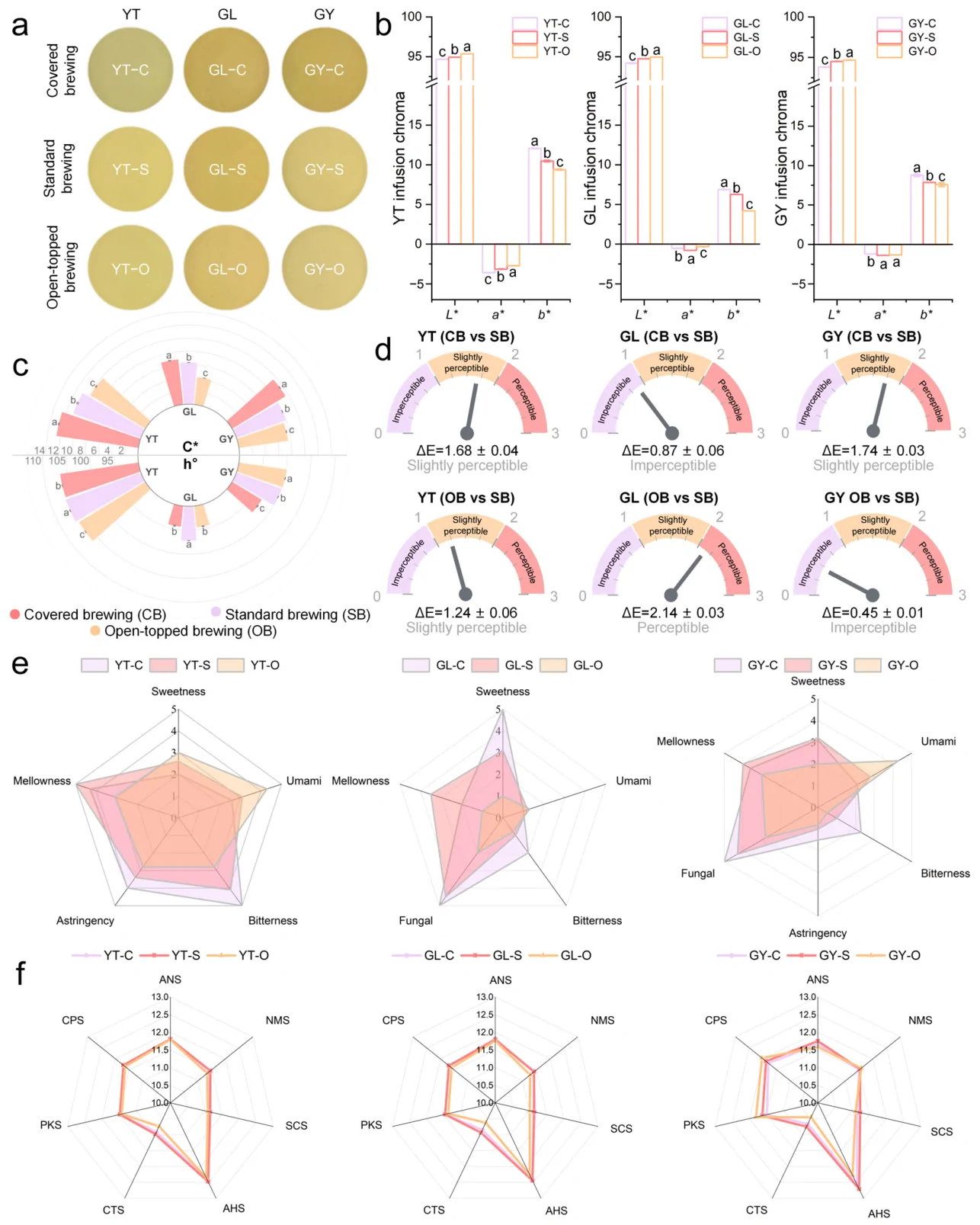
Effects of brewing methods on infusion appearance, color parameters, and sensory quality of YT, GL, and GY. (**a**) Infusion appearance; (**b**) color parameters (L*, a*, and b*); (**c**) chroma (C*) and hue angle (*h*°); (**d**) color difference (Δ*E*) relative to standard brewing; (**e**) taste evaluation; (**f**) electronic tongue response. YT-S, YT-C, and YT-O, yellow tea infusions prepared using standard, covered, and open-topped brewing, respectively; GL-S, GL-C, and GL-O, *Ganoderma lucidum* infusions prepared using standard, covered, and open-topped brewing, respectively; GY-S, GY-C, and GY-O, *Ganoderma lucidum* yellow tea infusions prepared using standard, covered, and open-topped brewing, respectively. ANS, sweetness sensor; NMS, umami; SCS, bitterness sensor; AHS, sourness sensor; CTS, saltiness sensor; PKS and CPS, general-purpose sensors. Sensory scores are presented as the mean values obtained from seven panelists (*n* = 7). Different lowercase letters indicate significant differences among brewing methods within the same sample group (*p* < 0.05).

Brewing method is an important external factor governing the quality expression of flavored tea because it determines heat retention, extraction intensity, and gas exchange during infusion, thereby influencing the aroma and taste presentation [7]. For example, vacuum-insulated brewing, characterized by stronger heat retention and more restricted oxygen exchange, has been reported to induce a stewed off-flavor in green tea [32]. Brewing factors can alter the release of taste-active and color-related compounds, thereby influencing infusion appearance and taste coordination. As shown in Figure 2a, YT infusion appeared bright yellow, while GL infusion presented as a darker orange-yellow. GY also displayed a deeper orange-yellow color than YT while lighter than GL, indicating that the addition of GL altered the infusion color of YT. The brewing method further modulated brightness and chromaticity. Covered brewing (CB) produced the darkest infusion, open-topped brewing (OB) produced the lightest infusion, and standard brewing (SB) showed an intermediate appearance. These visual differences were confirmed by colorimetric analysis (Figure 2b). Compared with SB, CB consistently decreased L* and increased b* and C*, indicating darker, more yellow, and more chromatic infusions (Figure 2c). Specifically, C* increased from 10.94 to 12.60 for YT, from 6.31 to 6.89 for GL, and from 7.97 to 9.51 for GY. In contrast, OB increased L* but reduced b* and C*, resulting in lighter and less chromatic infusions. The hue angles remained within 94.36–106.74°, indicating that the infusions retained their basic yellow to yellow-green hue, although CB shifted *h*° toward the yellow direction, particularly for GL and GY. Relative to SB, the Δ*E* values of CB and OB were 1.68 and 1.24 for YT, 0.87 and 2.14 for GL, and 1.74 and 0.45 for GY, respectively (Figure 2d). The largest color difference was observed for GL prepared by OB, whereas GY prepared by OB showed the smallest difference. Overall, these results demonstrate that brewing method significantly influenced infusion color and that the magnitude of this effect depended on the tea material.

Brewing method also markedly changed the taste profile (Figure 2e). CB enhanced the fungal sensory characteristics of GY, but also intensified bitterness and astringency, resulting in relatively insufficient umami and sweetness. In contrast, OB was more conducive to highlighting umami and reduced bitterness and astringency to some extent, although its effect on promoting the characteristic flavor of GL was limited. SB enhanced the mellow taste of GY while producing lower bitterness and astringency than CB, indicating better taste coordination. This may be attributed to the fact that SB ensured sufficient extraction of taste-related compounds while avoiding excessive release of bitter and astringent substances. Previous studies have shown that a higher brewing temperature facilitates the extraction of internal constituents, whereas excessively high temperatures promote the release of catechins into tea infusions, thereby intensifying bitterness and astringency [33]. Therefore, SB may promote a balanced release of umami- and sweet-tasting compounds as well as bitter and astringent components through appropriate temperature maintenance, ultimately contributing to a more harmonious taste profile.

The electronic tongue further distinguished the samples (Figure 2f). For YT samples, the differences in sensor responses caused by the three brewing methods were relatively small. For GL samples, only the OB-brewed sample (GL-O) exhibited a distinct response pattern, with weaker responses across all sensors than the SB- and CB-brewed samples (GL-S and GL-C). In contrast, GY was more sensitive to brewing methods, showing clear differences among the different brewing conditions. Specifically, GY brewed using SB (GY-S) exhibited higher responses in the sweetness sensor (ANS), bitterness sensor (SCS), sourness sensor (AHS), and saltiness sensor (CTS), indicating that SB promoted the sufficient extraction of compounds associated with multiple electronic tongue taste responses in GY. The overall response pattern of GY brewed using CB (GY-C) was similar to that of GY-S, although with slightly lower response intensities. By contrast, GY brewed using OB (GY-O) showed the lowest responses in most sensors. These results suggested that SB may be more favorable than CB and OB for the extraction of compounds associated with electronic tongue taste responses in GY. Notably, the sensory evaluation and electronic tongue results were consistent in terms of the sweetness quality of GY-S, both showing higher levels than those of GY-C and GY-O. However, discrepancies were observed between the two evaluation methods for other taste attributes. This difference may arise because human sensory perception is not a simple linear summation of individual taste signals, but is comprehensively regulated by the interactions among multiple taste and aroma compounds. For example, previous research has shown that the addition of volatile compounds such as octanol, cis-3-hexenyl acetate, and furaneol to Sichuan green tea can mask bitterness, thereby influencing the overall perception of tea infusion taste quality [34]. In contrast, the electronic tongue mainly collects electrochemical signals through a sensor array and converts them into digital responses. It therefore primarily reflects the response intensity of taste-related substances in the liquid phase that can be recognized by the sensors, but cannot fully simulate the integrated interactions among aroma, taste, and texture involved in human oral perception. Accordingly, electronic tongue analysis can serve as an important complement to sensory evaluation, but should not be regarded as fully equivalent to human sensory experience.

Overall, Figure 2 demonstrated that brewing method regulated both infusion color and sensory balance in the optimized GY. This is consistent with previous studies showing that raw material blending and brewing parameters jointly shape tea infusion quality by modulating sensory expression [1,32]. Covered brewing produced a darker infusion and intensified bitterness, astringency, and fungal notes, suggesting that extraction rate was elevated under persistent heat and high pressure. Color-related and undesirable taste-active compounds were released simultaneously. Meanwhile, chemical transformation such as isomerization and hydrolysis occurred [5]. This result agreed with the reported formation of stewed off-flavor in vacuum-insulated green tea brewing under strong heat retention and restricted gas exchange [32]. By contrast, open-topped brewing yielded a lighter infusion with stronger umami but weaker sweetness, mellowness, and fungal flavor, indicating insufficient extraction of compounds responsible for flavor fullness. Thus, SB may provide the optimal extraction balance, enhancing desirable taste attributes while limiting excessive transformation and extraction of undesirable compounds. The magnitude of the brewing-dependent sensory differences was greater for GY than for YT, indicating that the response to brewing depended on the composition of the infusion matrix. Theoretically, the blend ratio determines the relative abundance of YT-and GL-derived flavor-active and particle-forming precursors, whereas brewing governs their extraction, transformation, volatilization, and phase partitioning. The observed sensory profile therefore reflects the specific formulation-brewing combination rather than an isolated effect of either factor. Nevertheless, because all blend ratios were not examined under all three brewing methods, this matrix dependence remains descriptive and should be confirmed using a full factorial design.

### 3.3. Chemical Components of the Ganoderma lucidum Yellow Tea Samples with Different Brewing Methods

**Figure 3:**
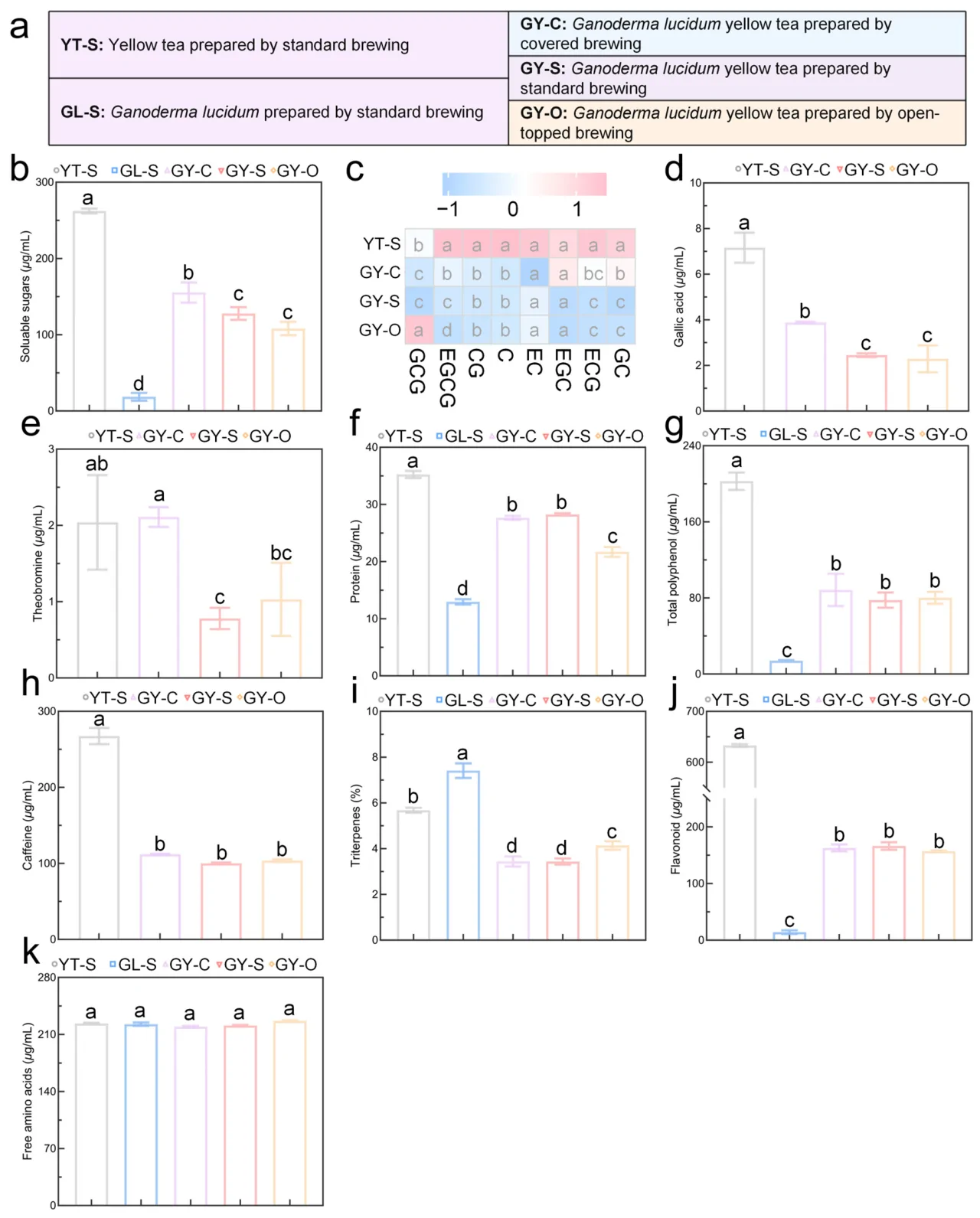
Effects of brewing methods on the major biochemical components of GY. (**a**) abbreviation example; (**b**) soluble sugars; (**c**) heatmap of individual catechins, including GCG, EGCG, CG, C, EC, EGC, ECG, and GC, with their detailed concentrations provided in Table S1; (**d**) gallic acid; (**e**) theobromine; (**f**) protein; (**g**) total polyphenols; (**h**) caffeine; (**i**) triterpenoids; (**j**) flavonoids; (**k**) free amino acids. YT-S, yellow tea prepared by standard brewing; GL-S, *Ganoderma lucidum* prepared by standard brewing; GY-C, GY-S, and GY-O, *Ganoderma lucidum* yellow tea prepared by covered, standard, and open-topped brewing, respectively. C, catechin; GC, gallocatechin; CG, catechin gallate; GCG, gallocatechin gallate; EC, epicatechin; EGC, epigallocatechin; ECG, epicatechin gallate; EGCG, epigallocatechin gallate. Different lowercase letters indicate significant differences among brewing methods within the same sample group (*p* < 0.05).

Taste quality is determined by the composition and relative proportions of water-extractable compounds [9]. To explain the brewing-dependent sensory quality differences, the composition of catechins and contents of flavonoids, tea polyphenols, proteins, and soluble sugars were measured in GY samples prepared by different brewing methods, with YT brewed using SB (YT-S) and GL-S used as controls. Soluble sugars are important compounds contributing to sweetness in tea infusions [35]. The soluble sugar content in GY-C was significantly higher than that in GY-S and GY-O, indicating that CB promoted the extraction of soluble sugars from GY (Figure 3b). However, GY-C did not show the greatest perceived sweetness despite its higher soluble-sugar content and instead exhibited pronounced bitterness and astringency. This finding departs from the simple expectation that higher sugar concentrations would increase sweetness [35] and indicates that observations for individual taste-active compound classes cannot be directly extrapolated to the present multicomponent infusion. Interactions among taste-active and colloidal constituents may therefore be more important than the concentration of any single analyte [36,37,38,39]. EGCG is the major catechin responsible for the strong bitterness and astringency of tea [37]. Its content was significantly higher in GY-C than in GY-S and GY-O, which may partly explain the stronger bitter and astringent taste of GY-C (Figure 3c). In addition, gallic acid and theobromine, both of which can enhance bitterness in tea infusions [38,39], were also present at significantly higher levels in GY-C than in GY-S and GY-O (Figure 3d,e). The higher levels of EGCG, gallic acid, and theobromine coincided with stronger bitterness and astringency in GY-C and may have contributed to this sensory pattern. However, these associations do not demonstrate individual or synergistic causality, because perceived bitterness and astringency reflect interactions among multiple taste-active and matrix constituents. Although temperature-dependent extraction of these compounds has been reported [40,41,42], the present data do not establish heat retention as the mechanism underlying their higher concentrations. Therefore, although GY-S and GY-O contained significantly lower levels of soluble sugars than GY-C, their lower levels of bitter and astringent compounds may have reduced the masking effect on sweetness.

Notably, brewing can also affect the extraction of flavor compounds of tea infusions. Proteins, caffeine, and polyphenols are important constituents exert flavor-related effects [43]. For instance, proteins can bind to salivary mucins, thereby affecting oral friction and astringency, whereas caffeine and polyphenols are closely associated with the bitter and astringent taste of tea infusions [36,44]. The results showed that the protein contents in GY-C and GY-S were significantly higher than that in GY-O (Figure 3f). In contrast, no significant differences were observed in the contents of polyphenols and caffeine among tea infusions prepared using different brewing methods, suggesting that brewing method had a limited effect on the extraction of these two compounds (Figure 3g,h). Triterpenoids are among the major pharmacologically active constituents of *Ganoderma lucidum* and are generally associated with a slightly bitter or bitter taste [28]. Comparison among different brewing methods showed that the triterpenoid content was significantly higher in GY-O, which had the weakest heat-retention effect and the lowest headspace pressure, than in GY-C and GY-S (Figure 3i). This phenomenon may be related to the relatively low thermal stability of triterpenoids. Previous studies have shown that, during the extraction of ganoderic acids from *Ganoderma lucidum*, an appropriate increase in extraction temperature can improve extraction efficiency. However, when the extraction temperature exceeds 75 °C, ganoderic acids may undergo rapid degradation [45]. In contrast, the contents of flavonoids and free amino acids showed only minor differences among the different brewing methods (Figure 3j,k). Furthermore, YT-S contained significantly higher levels of soluble sugars, catechins, gallic acid, proteins, polyphenols, caffeine, flavonoids, and several other compounds than all other samples, highlighting the dominant contribution of yellow tea to the taste profile of the flavored tea system. With the exception of triterpenoid compounds, which were significantly more abundant in GL-S than in the other samples, the levels of all other compound classes were relatively low. Moreover, catechins, gallic acid, theobromine, and caffeine were not detected in GL-S.

Overall, brewing method significantly affected the extraction and transformation of the chemical constituents of GY. Owing to its stronger heat-retention effect, CB showed the highest extraction efficiency for multiple compounds. However, excessive extraction of bitter and astringent substances disrupted the flavor balance of GY. OB, which had the weakest heat-retention effect, resulted in the lowest extraction levels of many compounds, potentially explaining the relatively weak overall taste of GY. By contrast, SB achieved the best balance between compound extraction and bitter-astringent taste, thereby producing the most favorable flavor profile of GY.

### 3.4. Colloidal Particle Characteristics of Ganoderma lucidum Yellow Tea Prepared by Different Brewing Methods

**Figure 4:**
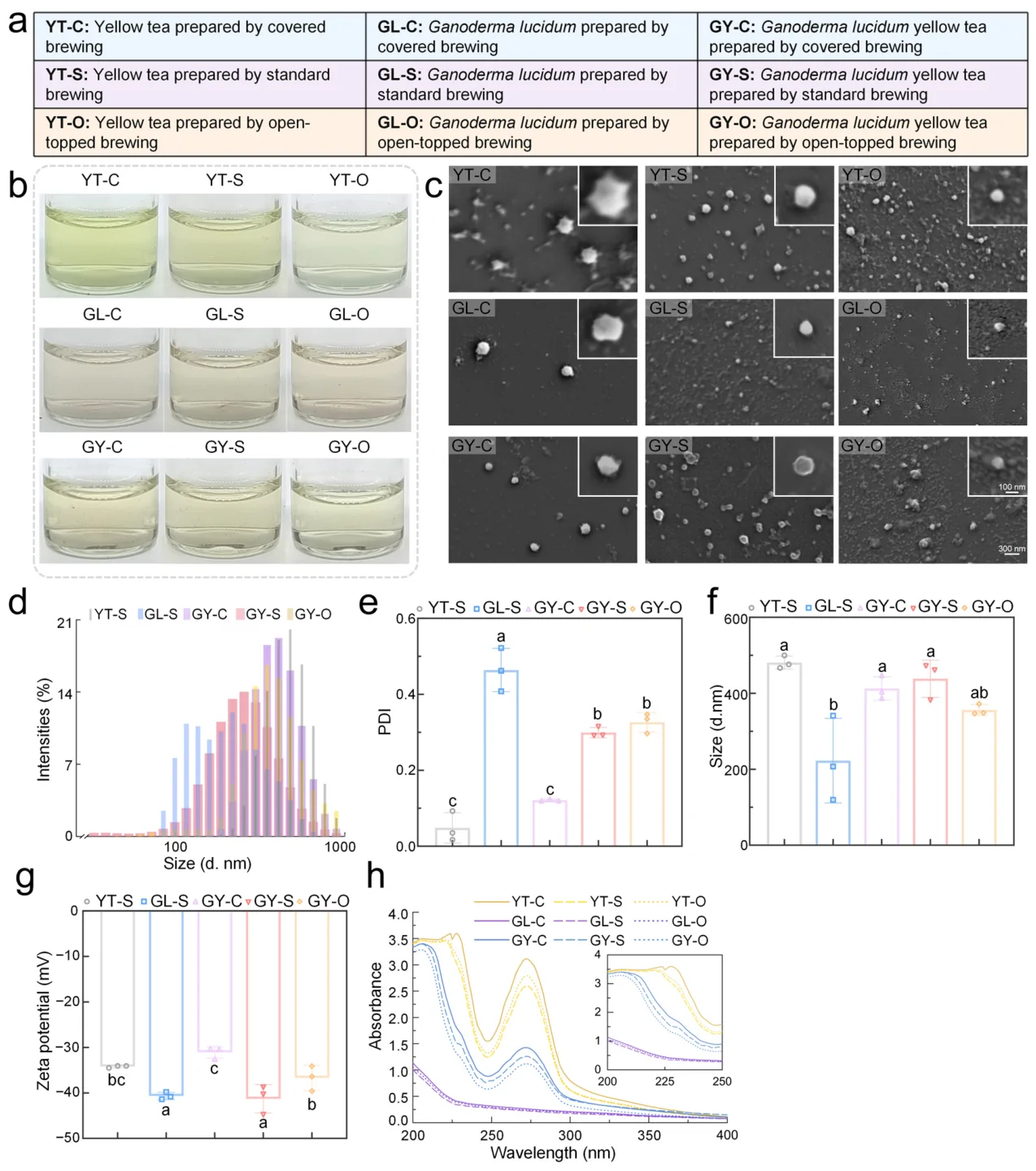
Effects of brewing methods on the properties of colloidal particles in GY. (**a**) abbreviation example; (**b**) photographs of infusions; (**c**) scanning electron microscopy images of colloidal particles; (**d**) particle size distribution; (**e**) polydispersity index (PDI); (**f**) average particle size; (**g**) zeta potential; (**h**) UV-vis absorption spectra. YT-S, YT-C, and YT-O, yellow tea infusions prepared using standard, covered, and open-topped brewing, respectively; GL-S, GL-C, and GL-O, *Ganoderma lucidum* infusions prepared using standard, covered, and open-topped brewing, respectively; GY-S, GY-C, and GY-O, *Ganoderma lucidum* yellow tea infusions prepared using standard, covered, and open-topped brewing, respectively. Different lowercase letters indicate significant differences among samples (*p* < 0.05).

Tea infusion is a complex dispersed system rather than a simple true solution, containing small taste-active molecules, proteins, polyphenols, and their supramolecular self-assembled [43,46]. Apart from flavor-related compounds, natural infusion NPs also contributed to mouthfeel including roughness, mellowness, etc. [27]. As shown in Figure 4b, NPs-enriched infusion of YT brewed using CB (YT-C), YT-S and YT brewed using OB (YT-O) appeared yellow-green, light yellow-green, and pale yellow-green, respectively. The color of GL colloid infusion presented similar nearly colorless appearance. Among the different brewing methods, GY-C colloidal infusion exhibited the deepest beige-yellow color, whereas GY-O showed the lightest color. For colloidal dispersions assembled from similar material constituents, a deeper color generally indicates a higher relative abundance of colloidal particles [47]. Accordingly, consistent with the material basis of these colloidal systems, GY-C contained the highest relative abundance of NPs, whereas GY-O contained the lowest. The surface morphology and size were reckoned as crucial factors in mellowness [14,48]. The morphological characteristics of NPs from different samples were observed by SEM (Figure 4c). Comparison among brewing methods showed that GY-C NPs had more irregular edges than GY-S NPs, with small particles aggregated around the larger particles. Previous studies have shown that particles with rougher surfaces are more readily perceived during oral processing and can induce grittiness [49]. Compared with GY-C and GY-S, GY-O NPs were substantially smaller overall. YT NPs manifested as spherical or near-spherical shape. GL NPs presented much smaller size and accompanied by plentiful ultra-tiny NPs (attached and around). Consistent with the SEM image, particle size distribution differed substantially among samples with different brewing methods (Figure 4d).

The particle size distribution of GY-C was more concentrated than those of GY-S and GY-O, which was consistent with the polydispersity index (PDI) results (Figure 4e). A lower PDI indicates a narrower particle size distribution [50]. These results suggest that CB promoted the concentrated aggregation and formation of GY NPs. Interestingly, although the main peak of the GY-S particle size distribution was located at 254.37 nm, which was lower than that of GY-C at 407.16 nm, the peak value of GY-S was also lower than that of GY-C, with values of 14.02% and 19.04%, respectively. However, the average particle size of GY-S was comparable to that of GY-C, with its mean value being even slightly higher. This may be attributed to the broader particle size range and more even distribution of GY-S, as reflected by its significantly higher PDI than GY-C, which increased the calculated mean particle size (Figure 4f). Larger particles can produce higher perceptual intensity [49]. Together with the relatively smooth surface morphology of GY-S NPs, this may contribute to the formation of a smoother mouthfeel in GY-S. In addition, ultra-small NPs beneath 150 nm were abundant in the GL infusion but were scarcely detected in the GY colloidal infusion. It indicated that the brewing of flavored tea is not equal to the mixture of raw material infusion, but a self-assembly process to form novel types of NPs.

Zeta potential is an important indicator for characterizing the surface charge properties and electrostatic stability of colloidal particles [51]. The zeta potentials of NPs from all samples were negatively charged (Figure 4g), which may be attributed to the abundant hydroxy and carboxyl groups from polyphenols, proteins, etc. [52]. The absolute value of GY samples is relatively high (>30 mV), indicating the potential high stability. Comparison among brewing methods showed that GY-S had the highest absolute zeta potential, whereas GY-C had the lowest. NPs are mainly assembled through interactions among tea polyphenols, proteins, polysaccharides, caffeine, and other constituents in the dissolved phase of the infusion. Therefore, differences in zeta potential are largely influenced by the chemical composition of the original infusion. Previous studies have shown that catechins such as EGCG and ECG readily interact with caffeine (CAF), resulting in a significant decrease in the absolute zeta potential value of the formed complexes [51]. In the present study, the EGCG content in the original GY-C infusion was significantly higher than those in GY-S and GY-O. The EGC content was also higher in GY-C than in GY-S and GY-O. These parallel changes are consistent with a possible contribution of catechin-caffeine interactions to the lower absolute zeta potential of GY-C NPs. However, the presence and structures of EGCG-CAF and EGC-CAF complexes in these particles were not directly confirmed in the present study. Significant differences in UV absorption of YT, GL, and GY indicated the distinguished surface chemical basis (Figure 4h). YT samples showed absorption peaks at 220 nm for YT-S and YT-O, and at 228 nm for YT-C. By contrast, the UV absorbance of GY decreased rapidly after 206 nm. This phenomenon may result from the combined contributions of UV-active components, including peptide bonds, carbonyl groups, catechins, phenolic acids, and caffeine [53,54]. Theoretically, triterpenoid compounds should bring about the absorption at around 235 nm, whose absence may be ascribed to the embedding or masking of NPs structure [55].

Overall, brewing method markedly influenced the colloidal characteristics of GY infusion. CB promoted the aggregation of GY NPs, resulting in the deepest colloidal color, larger and more concentrated particle size distribution, rougher particle edges, and the lowest absolute zeta potential, which may be associated with stronger bitterness, astringency, and grittiness. In contrast, SB produced GY NPs with a broader particle size distribution, relatively smooth morphology, and the highest absolute zeta potential, indicating better colloidal stability and a potential contribution to smoother and mellower mouthfeel. OB generated smaller particles and the lowest relative abundance of NPs, corresponding to a weaker colloidal structure and flatter taste. Therefore, the sensory quality of GY infusion was closely associated with brewing-induced changes in NP abundance, morphology, size distribution, surface charge, and chemical structure.

### 3.5. Chemical Characteristics of Nanoparticles from Flavored Tea Prepared by Different Brewing Methods

**Figure 5:**
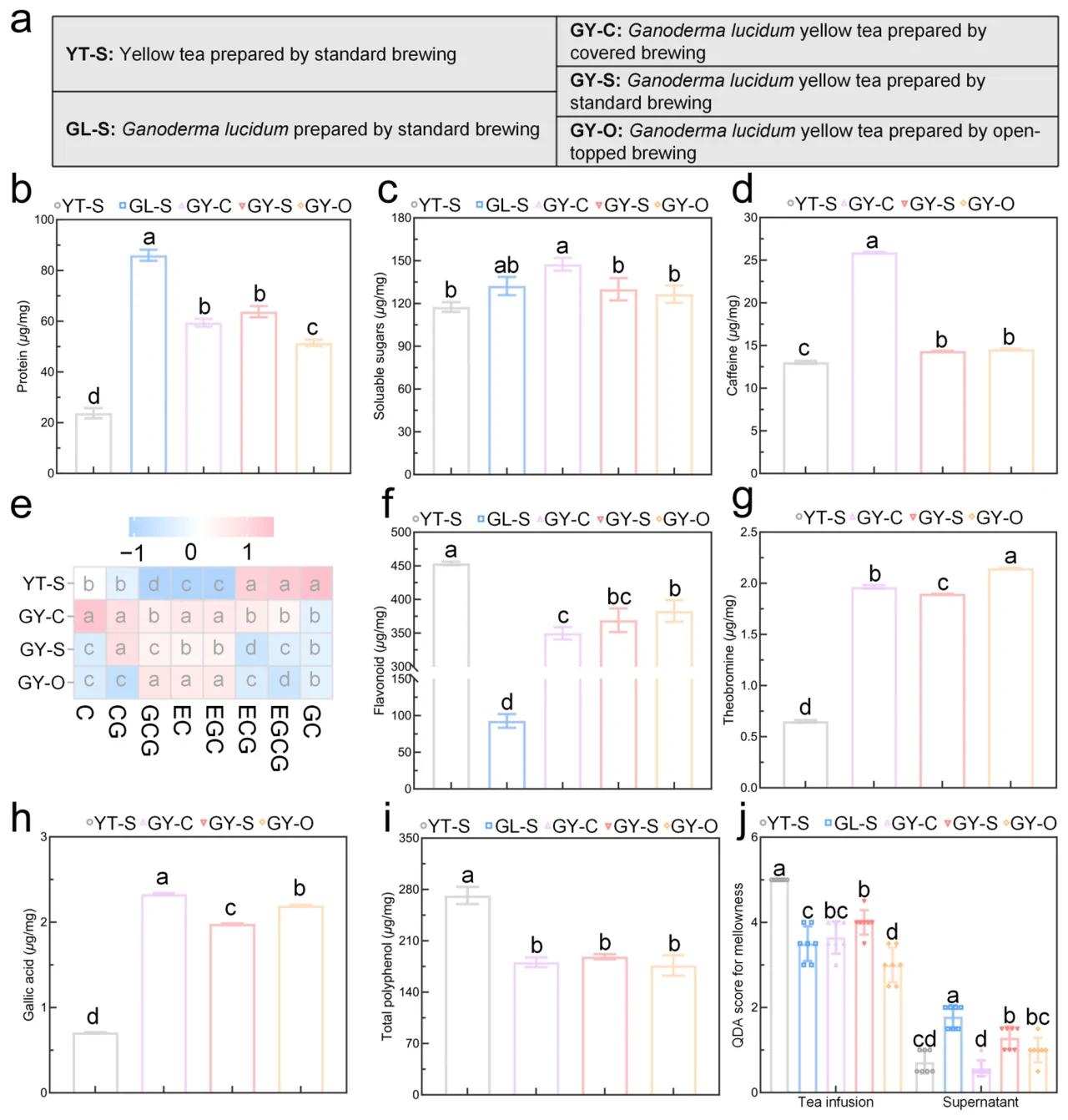
Chemical basis of colloidal particle assembly in GY. (**a**) abbreviation example; (**b**) protein; (**c**) soluble sugars; (**d**) caffeine; (**e**) heatmap of individual catechins, including GCG, EGCG, CG, C, EC, EGC, ECG, and GC, with their detailed concentrations provided in Table S2; (**f**) flavonoids; (**g**) theobromine; (**h**) gallic acid; (**i**) total polyphenols; (**j**) QDA score for mellowness. YT-S, yellow tea prepared by standard brewing; GL-S, *Ganoderma lucidum* prepared by standard brewing; GY-C, GY-S, and GY-O, *Ganoderma lucidum* yellow tea prepared by covered, standard, and open-topped brewing, respectively. Different lowercase letters indicate significant differences among samples (*p* < 0.05).

To further elucidate the contribution of NPs to taste and mouthfeel, the chemical basis of NPs was analyzed. Previous research has demonstrated that proteins and soluble sugars represent important macromolecular components in colloidal particle formation [12]. The protein contents of YT-S and GL-S in NPs showed an opposite trend to that observed in the original tea infusions (Figure 5b). In the original infusions, YT-S exhibited a significantly higher protein content than the other samples, whereas in NPs, GL-S showed a significantly higher protein content than the other samples. This result indicates that, compared with proteins derived from yellow tea, proteins derived from *Ganoderma lucidum* may be more favorable for assembly and migration from the true solution phase to the colloidal fraction. This phenomenon may be related to protein type and structural characteristics. For example, previous studies have shown that in milk tea systems, casein tends to bind preferentially to high-molecular-weight tea polyphenols, whereas whey protein shows a higher affinity for low-molecular-weight tea polyphenols [43]. In addition, proteins with more exposed hydrophobic regions and higher proportions of aromatic residues, such as tryptophan and tyrosine, are more likely to interact with tea polyphenols and caffeine, thereby forming complexes [43]. Because YT-S lacked sufficient protein as the core structural component of the colloidal fraction, the assembly-related molecules, including soluble sugars, caffeine, catechins, and gallic acid, also showed trends opposite to those in the original infusion, with lower contents than those in the GY samples.

By contrast, the changing trends of proteins and soluble sugars in GY NPs prepared using different brewing methods were largely consistent with those observed in the original infusions (Figure 5c). The protein contents of GY-C and GY-S were significantly higher than that of GY-O, and the soluble sugar content of GY-C was significantly higher than those of GY-S and GY-O. These results suggest that brewing method exerted a relatively limited effect on the distribution of macromolecular components between the liquid phase and the colloidal phase. In contrast, small molecules, including caffeine, catechins, and flavonoids, showed trends distinct from those in the original infusions. Although caffeine contents were relatively similar among the original infusions prepared using different brewing methods, the caffeine content in GY-C NPs was significantly higher than those in GY-S and GY-O NPs, indicating that CB promoted the migration of caffeine from the liquid phase to the colloidal phase (Figure 5d). Caffeine has a strong binding capacity and can form complexes with EC, EGC, ECG, and EGCG at stoichiometric ratios of 1:1, 1:1, 4:2, and 2:2, respectively, mainly through hydrogen bonding, π–π interactions, and CH–π interactions [51]. Accordingly, for catechins, the differences among several catechin components were more pronounced in the colloidal fraction than in the liquid phase under CB (Figure 5e). In particular, ECG and EGCG contents were significantly higher in GY-C than in GY-S and GY-O. Unlike the liquid phase, in which no significant difference was observed, the flavonoid contents of NPs differed significantly among the GY samples (Figure 5f). GY-O exhibited the highest flavonoid content, whereas GY-C showed the lowest flavonoid content, further confirming the regulatory effect of brewing method on the self-assembly process of NPs. Theobromine showed a distinct brewing-dependent distribution in the colloidal phase, with GY-O NPs containing the highest level among the GY samples, whereas GY-S NPs contained the lowest (Figure 5g). The gallic acid content also differed significantly among the GY colloidal fractions, decreasing in the order GY-C, GY-O, and GY-S (Figure 5h). By contrast, total polyphenol contents did not differ significantly among GY-C, GY-S, and GY-O NPs (Figure 5i).

In the present study, QDA analysis of the supernatants after NP removal also revealed a decrease in mellowness (Figure 5j). Among the samples, YT-S showed the most pronounced reduction in mellowness, which may be attributed to the largest particle size of YT-S NPs among the five samples. Although the lack of a protein-based core structure in YT-S NPs resulted in lower amounts of NP-enriched assembly components than in the other samples, previous studies have shown that EGCG–CAF and ECG–CAF can form NPs with considerably larger particle sizes than EC–CAF and EGC–CAF complexes [51]. In the present study, EGCG and ECG were significantly enriched in YT-S NPs, thereby promoting the formation of larger particles. Larger particles are generally more perceptible in the oral cavity [49]. Therefore, removal of NPs resulted in the greatest reduction in mellowness in YT-S. In contrast, GL-S NPs had the smallest particle size, which may explain why GL-S showed the smallest decrease in mellowness after NP removal compared with the other samples. The decrease in mellowness after removal of the NP-enriched fraction is directionally consistent with Deng et al. [48], who associated a membrane-retained macromolecular fraction with the mellow and thick taste of Pu-erh tea. However, their study used membrane fractionation in a fermented tea system, whereas the present study used centrifugation-based removal in a YT-GL blend. This convergence supports a broader contribution of colloidal material to mouthfeel but does not imply an identical composition or mechanism.

Overall, NPs served as supramolecular carriers that integrated macromolecular components, mainly proteins and soluble sugars/polysaccharides, with small molecules, including caffeine, catechins, gallic acid, and flavonoids. GL-derived proteins were more readily incorporated into the colloidal fraction than YT-derived proteins, suggesting their stronger contribution to NP formation. Brewing method had a limited effect on the distribution of macromolecules between the liquid and colloidal phases, but markedly regulated the enrichment of small molecules. CB promoted the incorporation of caffeine, ECG, and EGCG into GY NPs, whereas OB favored flavonoid enrichment. After NP removal, mellowness decreased in all samples, and the greatest reduction occurred in YT-S. These findings confirm that NPs are key colloidal contributors to the mellow mouthfeel of yellow tea—*Ganoderma lucidum* infusions.

Beyond compositional effects, the present findings highlight brewing as a practical lever for flavor design in tea-based beverages. Brewing temperature, time, and method govern the extraction kinetics and relative proportions of taste-active compounds and have long been used to tailor the sensory profile of tea infusions [40,41,42]. Our results extend this understanding from the molecular level to the colloidal level: brewing method exerted a limited effect on the distribution of macromolecules between the liquid and colloidal phases, but it selectively regulated the enrichment of small taste-active molecules within NPs, with CB promoting the incorporation of caffeine, ECG, and EGCG and OB favoring flavonoid enrichment. This means that brewing parameters can be exploited not only to control what is extracted but also how the extracted compounds are organized and presented in the cup. Such dual regulation is relevant to the industrial production of tea beverages. In ready-to-drink tea processing, uncontrolled particle self-assembly leads to creaming, haze, and sediment, which compromise appearance, taste, and shelf stability [43]. Conversely, deliberately programmed extraction could steer NP assembly toward desirable flavor outcomes while suppressing undesirable precipitation. Supporting this view, the particle size and chemical composition of black tea infusion NPs were shown to evolve systematically with brewing time [12], and the colloidal architecture of black tea infusion could be reshaped through simple physicochemical adjustments such as pH [56]. Integrating such colloid-based insights into extraction and formulation design may therefore facilitate the development of standardized, flavor-targeted production technologies for GY and related tea beverages.

The particle-removal experiments further position NPs as active sensory contributors rather than inert turbidity. NP removal decreased mellowness in all samples, with the greatest reduction observed in YT-S. A previous sensory-guided study on ripe Pu-erh tea similarly showed that mellow and thick taste was mainly associated with macromolecular and colloidal fractions rather than with small molecules alone [48]. Because larger particles are more readily perceived in the oral cavity [49], the size and morphology of infusion NPs represent structural variables through which mouthfeel can be modulated. Recent colloid-focused studies reinforce this view. The mean particle size of black tea infusion NPs increased progressively with brewing time, accompanied by dynamic changes in the incorporation of caffeine, catechins, proteins, and soluble sugars into the assembled fraction [12]. The micro- and nanostructure of black tea infusion could be disassembled or aggregated by adjusting pH [56]. Natural NPs in white tea infusion were shown to evolve in composition during aging, in parallel with changes in infusion quality [27], and their composition–structure–stability relationships were closely associated with haze formation and flavor performance [57]. Taken together, these findings indicate that the sensory contribution of tea infusion NPs is not fixed but can be rationally regulated through brewing time, brewing method, and the physicochemical environment of the infusion. Steering the formation, size, morphology, and composition of infusion NPs thus represents a promising direction for the precise modulation of mellow mouthfeel and overall flavor in tea beverages.

Although these findings offer an integrated perspective on how formulation and brewing relate to the sensory quality of GY, several limitations should be considered. Although targeted chemical profiling and particle-removal experiments supported associations among chemical composition, colloidal organization, and sensory quality, they did not resolve the underlying molecular mechanisms. The broader metabolomic profile was not characterized; therefore, the chemical basis of the observed sensory differences may not have been fully captured. Future studies should validate these findings through factorial designs, independent material batches, consumer testing, comprehensive metabolomic profiling, native-state colloidal characterization, and assessments of process scale-up and shelf stability.

## 4. Conclusions

This study advances current understanding of flavored-tea quality by indicating that the sensory performance of a medicinal mushroom-tea blend cannot be interpreted from the total amount of extracted compounds alone, which is also associated with the balance and partitioning between dissolved and self-assembled colloidal phases. The principal theoretical implication is that brewing is a critical process variable that modulates the extraction, phase partitioning, and colloidal assembly of formulation-derived constituents, thereby influencing the sensory quality of the final infusion. Under the conditions tested, the 2:3 YT:GL formulation prepared by standard brewing provided the most coordinated sensory profile, balancing the characteristic *Ganoderma lucidum* aroma with sweetness, mellowness, bitterness, and astringency. The brewing method also reorganized the colloidal phase. Covered brewing promoted more concentrated particle aggregation, produced more irregular particles, and enhanced the incorporation of caffeine, ECG, and EGCG into GY nanoparticles, whereas standard brewing yielded a broader particle-size distribution, smoother particle morphology, a higher absolute zeta potential, and a more balanced colloidal composition. Removal of the NP-enriched fraction decreased mellowness in all samples, supporting the role of infusion nanoparticles as active contributors to mouthfeel. Together, these findings show that brewing regulates sensory quality through the coupled control of compound extraction and colloidal assembly. Future studies should integrate volatile and non-targeted metabolomics, sensory reconstitution, native-state colloid characterization, and oral tribology to clarify how aroma compounds, non-volatile constituents, colloidal structures, and oral interactions jointly determine the sensory quality of the beverage.

## Figures and Tables

**Table 1:** Sensory evaluation of GY with different blending ratios.

Sample	Blending Ratio (Yellow Tea: *G. lucidum*)	Total Score	Infusion Color		Aroma		Taste	
			Description	Score	Description	Score	Description	Score
YT	1:0	80.00 ± 0.29 ^e^	Yellow and bright	80.00 ± 0.26 ^d^	Sour and dull	80.00 ± 0.29 ^f^	Pure, slightly sweet	80.00 ± 0.26 ^e^
GL	0:1	-	Orange-yellow and bright	-	Strong *G. lucidum* aroma	-	Weak taste and pasty mouthfeel	-
GY-1	3:1	80.80 ± 0.35 ^d^	Yellow and relatively bright	81.00 ± 0.52 ^e^	Slight fungal aroma, with evident tea aroma	81.60 ± 0.36 ^d^	Green and astringent	80.00 ± 0.53 ^e^
GY-2	2:1	85.10 ± 0.40 ^b^	Orange-yellow and relatively bright	82.80 ± 0.65 ^b^	*G. lucidum* and tea aromas, with evident sweet aroma	88.00 ± 0.47 ^a^	Moderately sweet and umami, slightly rough and green	83.20 ± 0.34 ^c^
GY-3	3:2	85.60 ± 0.35 ^ab^	Orange-yellow and relatively bright	84.00 ± 0.35 ^a^	Distinct *G. lucidum* aroma, with tea aroma; harmonious	87.40 ± 0.61 ^a^	Mellow, slightly green and *G. lucidum* flavor; harmonious	84.60 ± 0.31 ^b^
GY-4	2:3	86.00 ± 0.33 ^a^	Orange-yellow and relatively bright	83.20 ± 0.35 ^ab^	Distinct *G. lucidum* aroma, with tea and sweet aromas	85.60 ± 0.35 ^b^	Sweet and mellow, slightly umami, and harmonious	87.20 ± 0.49 ^a^
GY-5	1:2	83.20 ± 0.59 ^c^	Orange-yellow and relatively bright	81.00 ± 0.60 ^cde^	Strong *G. lucidum* aroma, with slight tea aroma	84.00 ± 0.52 ^c^	Mellow, slightly astringent, with weak tea flavor	83.20 ± 0.38 ^c^
GY-6	1:3	81.30 ± 0.56 ^d^	Orange-yellow and relatively bright	82.40 ± 0.53 ^bc^	Excessively strong *G. lucidum* aroma	81.00 ± 0.41 ^e^	Mellow, sweet, with excessively strong *G. lucidum* flavor and lacking tea flavor	81.20 ± 0.49 ^d^

Note: YT, yellow tea; GL, *Ganoderma lucidum*; GY, *Ganoderma lucidum* yellow tea; GY-1–GY-6, *Ganoderma lucidum* yellow tea with different blending ratios. The hyphen (-) indicates that no corresponding score was assigned. Sensory scores are presented as mean ± SD obtained from seven panelists (*n* = 7). Within each column, values without a common superscript letter differ significantly according to paired comparisons with Holm adjustment (*p* < 0.05).

## Data Availability

The original contributions presented in this study are included in the article/Supplementary Material. Further inquiries can be directed to the corresponding authors.

## Supplementary Materials

The following supporting information can be downloaded at: https://www.mdpi.com/article/10.3390/foods15162876/s1, Table S1. Catechin composition of yellow tea and *Ganoderma lucidum* yellow tea infusions prepared using different brewing methods. Table S2. Catechin composition of nanoparticles isolated from yellow tea and *Ganoderma lucidum* yellow tea infusions prepared using different brewing methods.
