# Supplementary material for: Blend-Brewing Optimization of Ganoderma lucidum Yellow Tea: Improving Palatability by Regulating Compound Release and Nanoparticle Assembly
Source: Foods. 2026 Aug 17;15(16):2876. doi: 10.3390/foods15162876 (PMC13512221; doi:10.3390/foods15162876)
Supplement: Supplementary file 1 [file foods-15-02876-s001.zip › foods-4439902-supplementary.pdf]

Supplementary Materials

Blend-brewing optimization of *Ganoderma lucidum* yellow tea: improving palatability by regulating compound release and nanoparticle assembly

Anran Yan [1,†], Weiyi Kong [1,†], Xinyu Feng [1], Ning Tang [1], Fangyuan Fan [1], Ping Chen [1], Binggan Lou [2], Qiang Chu [1,*], Ruili Zhang [3,*]

1 Tea Research Institute, Zhejiang University, Hangzhou 310058, China
2 State Key Laboratory of Rice Biology and Breeding, Ministry of Agriculture Key Laboratory of Molecular Biology of Crop Pathogens and Insects, Institute of Biotechnology, Zhejiang University, Hangzhou 310058, China
3 Xinjiang Production & Construction Group Key Laboratory of Agricultural Products Processing in Xinjiang South, College of Food Science and Engineering, Tarim University, Alar 843300, China
* Authors to whom correspondence should be addressed.
† These authors contributed equally to this work.

Table S1. Catechin composition of yellow tea and *Ganoderma lucidum* yellow tea infusions prepared using different brewing methods.

Catechin (μg/mL)	YT-S	GY-C	GY-S	GY-O
GC	23.93 ± 2.73[a]	15.35 ± 2.77[b]	1.12 ± 0.45[c]	3.06 ± 0.21[c]
ECG	0.94 ± 0.21[a]	0.60 ± 0.24[bc]	0.33 ± 0.01[c]	0.34 ± 0.03[c]
EGC	8.80 ± 5.11[a]	7.19 ± 4.75[a]	0.65 ± 0.04[a]	0.58 ± 0.15[a]
EC	3.20 ± 2.81[a]	1.08 ± 0.75[a]	1.86 ± 0.89[a]	1.92 ± 1.48[a]
C	0.65 ± 0.01[a]	0.00 ± 0.00[b]	0.00 ± 0.00[b]	0.00 ± 0.00[b]
CG	1.18 ± 0.36[a]	0.46 ± 0.07[b]	0.39 ± 0.08[b]	0.35 ± 0.03[b]
EGCG	78.90 ± 4.12[a]	35.57 ± 1.98[b]	25.09 ± 1.42[c]	18.71 ± 1.23[d]
GCG	5.69 ± 1.13[b]	2.80 ± 0.72[c]	1.11 ± 0.13[c]	10.95 ± 0.01[a]

Values are presented as mean ± SD (n=3). Different lowercase superscript letters within the same row indicate significant differences among samples. YT-S, yellow tea infusion prepared by standard brewing; GY-C, GY-S, and GY-O, *Ganoderma lucidum* yellow tea infusions prepared by covered, standard, and open-topped brewing, respectively; C, catechin; CG, catechin gallate; EC, epicatechin; ECG, epicatechin gallate; EGC, epigallocatechin; EGCG, epigallocatechin gallate; GC, gallocatechin; GCG, gallocatechin gallate. Different lowercase letters indicate significant differences among brewing methods within the same sample group ($P<0.05$).

Table S2. Catechin composition of nanoparticles isolated from yellow tea and *Ganoderma lucidum* yellow tea infusions prepared using different brewing methods.

**Catechin (μg /mg)**	**YT-S**	**GY-C**	**GY-S**	**GY-O**
GC	0.63 ± 0.00^{a}	0.00 ± 0.00^{b}	0.00 ± 0.00^{b}	0.00 ± 0.00^{b}
ECG	3.48 ± 0.03^{a}	3.17 ± 0.04^{b}	2.46 ± 0.01^{d}	2.57 ± 0.01^{c}
EGC	0.89 ± 0.02^{c}	2.21 ± 0.07^{a}	2.07 ± 0.01^{b}	2.19 ± 0.01^{a}
EC	1.01 ± 0.01^{c}	2.45 ± 0.06^{a}	2.21 ± 0.01^{b}	2.41 ± 0.01^{a}
C	0.65 ± 0.01^{b}	1.91 ± 0.01^{a}	0.00 ± 0.00^{c}	0.00 ± 0.00^{c}
CG	0.64 ± 0.00^{b}	1.98 ± 0.13^{a}	1.93 ± 0.03^{a}	0.00 ± 0.00^{c}
EGCG	6.56 ± 0.11^{a}	4.83 ± 0.09^{b}	3.34 ± 0.02^{c}	2.73 ± 0.03^{d}
GCG	0.80 ± 0.00^{d}	1.98 ± 0.02^{b}	1.91 ± 0.01^{c}	2.12 ± 0.02^{a}

Values are presented as mean ± SD (n=3). Different lowercase superscript letters within the same row indicate significant differences among samples. YT-S, yellow tea infusion prepared by standard brewing; GY-C, GY-S, and GY-O, *Ganoderma lucidum* yellow tea infusions prepared by covered, standard, and open-topped brewing, respectively; C, catechin; CG, catechin gallate; EC, epicatechin; ECG, epicatechin gallate; EGC, epigallocatechin; EGCG, epigallocatechin gallate; GC, gallocatechin; GCG, gallocatechin gallate. Different lowercase letters indicate significant differences among brewing methods within the same sample group ($P<0.05$).
